# Genotypic Characterization and Evaluation of *Japonica* Soft Rice Varieties in the Yangtze River Delta Region of China

**DOI:** 10.3390/cimb48070738

**Published:** 2026-07-20

**Authors:** Fuan Niu, Yuting Dai, Can Cheng, Anpeng Zhang, Huangwei Chu, Jihua Zhou, Bin Sun, Xiao Gu, Hua Wang, Kaizhen Xie, Fengzhen Shi, Xueqing Zhang, Bilian Hu, Yue Qiu, Xinyue Zhao, Wei Tian, Liming Cao

**Affiliations:** 1 Key Laboratory of Germplasm Innovation and Genetic Improvement of Grain and Oil Crops, Co-Construction by Ministry and Province, Ministry of Agriculture and Rural Affairs, Shanghai Academy of Agricultural Sciences, Shanghai 201403, China; niufuan@saas.sh.cn (F.N.); daiyuting@saas.sh.cn (Y.D.); chengcan@saas.sh.cn (C.C.); zhanganpeng@saas.sh.cn (A.Z.); chuhuangwei@saas.sh.cn (H.C.); zhoujihua@saas.sh.cn (J.Z.); sunbin@saas.sh.cn (B.S.); 18621311381@163.com (K.X.); 15987961804@163.com (F.S.); zxq12250314@163.com (X.Z.); bilianaya@163.com (B.H.); qiuyue0124@outlook.com (Y.Q.); 15251075879@163.com (X.Z.); tonywoody@163.com (W.T.); 2 Songjiang District Agricultural Technology Extension Center, Shanghai 201600, China; gx13sh@163.com; 3 Shanghai Municipal Agriculture Science and Technology Service Center, Shanghai 200335, China; m13052225542@163.com

**Keywords:** *japonica* soft rice, genotypic characterization, gene chip, functional gene, breeding

## Abstract

*Japonica* soft rice varieties possess excellent eating quality, and their cultivation area has been steadily expanding in recent years. This study aimed to analyze *japonica* soft rice varieties cultivated in the Yangtze River Delta region of China at the genome level and to provide a theoretical basis for optimizing disease resistance and other important traits. Genotypic characterization and evaluation of ten major *japonica* soft rice varieties from the Yangtze River Delta region were conducted using a genome-wide single nucleotide polymorphism (SNP) chip. The experimental results indicated that the soft rice varieties in the Yangtze River Delta region had a relatively high *japonica* component and were all classified as typical *japonica* rice varieties. Specifically, the highest (95.6%) and lowest (91.5%) proportions of *japonica* genomic segments were detected in Tai’an 1 and Zhehexiang 2, respectively. *Japonica* soft rice varieties from Shanghai exhibited a closer genetic distance to those from Jiangsu Province than to those from Zhejiang Province. Genomic identity was highest between Tai’an 1 and Nanjing 46 (87.9%) and lowest between Tai’an 1 and Jia 67 (74.4%). Based on the results of the chip assay, a total of twenty-six functional genes controlling key traits, such as yield, quality, and resistance to biotic and abiotic stresses, were identified in the ten analyzed varieties. Among them, Zhehexiang 2 carried the broad-spectrum blast resistance genes *Pi2* and *Pita*, which is useful for improving the blast resistance of *japonica* soft rice varieties. The findings of this study provide genetic resources and carrier materials for the efficient molecular improvement of *japonica* soft rice varieties.

## 1. Introduction

Rice (*Oryza sativa* L.) is the staple food for more than half of the global population, serving as a major source of nutrients and calories for humans. It is also the largest grain crop in China, accounting for 32% of the country’s total grain production [1]. Steady improvements in living standards have been accompanied by increasing demands for higher quality rice. Rice quality mainly encompasses milling quality, appearance quality, cooking and eating quality, and nutritional quality, of which cooking and eating quality is the most critical indicator. Eating quality is primarily influenced by the amylose content [2]. Generally, rice grains with excessive amounts of amylose (≥17%) absorb relatively large amounts of water during cooking, elongate poorly, and have a relatively high gelatinization temperature, resulting in a hard texture and poor taste. Conversely, rice with relatively little amylose (≤8%) yields incompletely expanded cooked grains that are too soft and sticky, bland in flavor, and generally unpalatable. By contrast, after cooking, *japonica* rice grains with an amylose content of 8–13% (i.e., *japonica* soft rice) are glossy, soft, elastic, and do not harden upon cooling, resulting in superior eating quality. Hence, their popularity among consumers has been increasing in recent years.

Since the 1980s, the premium-tasting rice market has grown rapidly worldwide, with many high-quality varieties bred internationally, among which varieties from Thailand and Japan represent a significant share of the global market. In China, soft rice varieties began to receive attention from breeders and researchers in the 21st century. The southwestern region of China, especially Yunnan, has long been used for the cultivation of soft rice varieties. The low amylose content of Yunnan soft rice grains is primarily due to *Wx^hp^* [3]. Over time, soft rice cultivation has gradually expanded to other regions, including Hunan and Hubei [4]. In the past decade, the breeding and subsequent cultivation of *japonica* soft rice varieties in the Yangtze River Delta region, including Jiangsu and Shanghai, has been vigorously and successfully promoted. The Institute of Food Crops, Jiangsu Academy of Agricultural Sciences, pioneered the use of *Wx^mp^* in molecular marker-assisted selection breeding, leading to the development of the Nanjing series of *japonica* soft rice varieties, including Nanjing 46, Nanjing 9108, and Nanjing 5055. In Shanghai, using Nanjing 46 as a soft rice parent, *japonica* soft rice varieties with different maturity periods, including Songzaoxiang 1, Huruan 1212, Songxiangjing 1018, and Tai’an 1, have been successively developed since 2014. From 2017 to 2023, a total of 86 rice varieties were approved in Shanghai, including 37 *japonica* soft rice varieties (43.0% of the approved rice varieties) [5]. Today, *japonica* soft rice is the dominant rice type produced in Shanghai.

While *japonica* soft rice varieties widely grown in the Yangtze River Delta region are characterized by superior palatability, they often display significant defects in appearance and disease resistance. Notably, weak disease resistance has become a key constraint hindering their broader adoption. Understanding the genetic information of rice varieties at the genomic level is crucial for advancing molecular improvement and germplasm innovation, particularly in terms of enhanced disease resistance. Low-throughput molecular markers represented by SSR have drawbacks such as a limited number of markers, low genome-wide coverage density, and time-consuming and labor-intensive detection, which imposes certain limitations in genomic breeding. The whole-genome single nucleotide polymorphism (SNP) chip for rice was developed on the basis of functional genomic research involving whole-genome sequencing and the subsequent screening for SNP markers closely associated with important agronomic traits, including disease resistance, insect resistance, grain quality and yield. The SNP chip is characterized by high marker density and uniform genome-wide coverage, making it important for gene identification and molecular breeding [6,7]. Ding et al. [8] effectively identified functional genes related to breeding in the Jiahe series varieties using the rice high-density chip GSR40K and clarified the characteristics and existing problems of Jiahe series varieties. Nevertheless, comprehensive genomic characterizations of *japonica* soft rice varieties cultivated in the Yangtze River Delta region are currently lacking. In this study, a high-density, genome-wide SNP array was employed to genotype and evaluate ten major *japonica* soft rice varieties from the Yangtze River Delta region, with the aim of providing a theoretical basis for the future molecular improvement and germplasm innovation of *japonica* soft rice.

## 2. Materials and Methods

### 2.1. Experimental Materials

The following ten major *japonica* soft rice varieties cultivated in the Yangtze River Delta region were selected as experimental materials: Songxiangjing 1018, Meigu 2, Huruan 1212, Tai’an 1, Nanjing 46, Nanjing 9108, Nanjing 5055, Zhehexiang 2, Jia 58, and Jia 67. Songxiangjing 1018, Meigu 2, and Tai’an 1 are the leading rice varieties grown in Shanghai. Huruan 1212 is the dominant rice variety in multiple districts of Shanghai, including Pudong New Area and Qingpu District, and was selected by several local rice brands as a dedicated variety for developing premium-tasting rice. Nanjing 46, Nanjing 9108, and Nanjing 5055 are the main promoted *japonica* soft rice varieties in Jiangsu Province, whereas Zhehexiang 2, Jia 58 and Jia 67 are the key *japonica* soft rice varieties promoted in Zhejiang Province. Experimental materials were grown at the Zhuanghang Comprehensive Experimental Station of Shanghai Academy of Agricultural Sciences (121.4° E, 30.9° N). Conventional water and fertilizer management practices were applied.

### 2.2. Analysis of Genetic Backgrounds

#### 2.2.1. Gene Chip-Based SNP Detection

SNPs were detected using the SLYm1R high-density rice whole-genome SNP chip by Wuhan Double Green Source Innovation Technology Research Institute Co., Ltd. (Wuhan, China). The chip, which was designed using Illumina chip manufacturing technology, contains 31,753 sites, with coordinate location information available for 31,704 of these sites; the remaining 49 sites were other probes. Filtering criteria for SNP markers included: (1) GenTrain score > 0.6; (2) Genotypes for parental lines were required to be homozygous (minimal heterozygosity < 5%); (3) call rate > 80%; (4) minor allele frequency(MAF) > 5%. The chip-based detection of SNPs involved the following two steps:

(1) Sample preparation

Genomic DNA was extracted from leaves collected at the rice tillering stage. After assessing DNA quality and determining that the DNA concentration exceeded 50 ng/µL, agarose gel electrophoresis was performed to ensure that the main DNA band was significantly larger than 5 kb. Finally, the DNA concentration was diluted to 50–100 ng/µL.

(2) Sample testing

DNA templates were denatured into single strands using NaOH, which was followed by whole-genome amplification. The amplified whole-genome DNA was then fragmented, after which DNA fragments were purified, resuspended, and denatured. The resulting DNA was applied to the designated positions on the chip, then the chip was placed in a hybridization oven at 48 °C for 16–24 h to complete the hybridization of DNA to the chip. Single base extension and staining were carried out on chip after hybridization. Finally, the chip was analyzed using the Illumina iScan^®^ system (San Diego, CA, USA), with the generated raw data analyzed and genotyped using the Illumina GenomeStudio^®^ software v2.0 (San Diego, CA, USA).

#### 2.2.2. Homozygosity Analysis

A rice chromosomal framework was constructed on the basis of the Nipponbare reference genome (version MSU7.0). High-quality loci revealed by the SLYm1R high-density rice whole-genome SNP chip were used along with the following formula to determine the homozygosity_ratio of test samples: homozygosity_ratio (%) = (number of homozygous loci/number of effective loci) × 100%.

#### 2.2.3. Analysis of *Indica*–*Japonica* Fragments

According to the genotyping results of the SLYm1R high-density rice whole-genome SNP chip, the rice genome was partitioned into bins of 200 kb each, with the remaining fragments < 200 kb at the 3′ ends of each chromosome counted as individual bins. A total of 1874 bins were generated, among which 1798 were suitable for *indica*–*japonica* genotyping. Typical *indica* varieties (Nanjing 11 and Nante) and *japonica* varieties (Balilla, Nipponbare, Youmangzaojing, and Nongken 58) were used as reference cultivars. Genome-wide markers in typical *japonica* varieties, but not in typical *indica* varieties, were extracted. Each bin was classified as either *indica* or *japonica*. The frequency of *indica* and *japonica* type genes was used to identify *indica* and *japonica* rice types. Classification criteria are presented in Table 1.

#### 2.2.4. Cluster Analysis

Pairwise SNP divergence was calculated across all samples to generate a divergence matrix. Divergence was defined as the proportion of mismatched genotypes at all loci between any two samples, with missing data excluded from the calculation. The resulting matrix was converted into a distance structure suitable for cluster analysis using the as.dist function in R (version 4.5.0). Hierarchical clustering was then performed on this distance matrix using the hclust function with the default complete-linkage method. Finally, the clustering result was transformed into a phylogenetic tree format using the ape package (version 5.8-1), and a downward-facing dendrogram was plotted using the plot function.

#### 2.2.5. Genomic Identity Analysis

Genomic identity was analyzed by dividing the rice genome into 378 bins (1 Mb each). If a SNP difference between samples was detected in a bin, the two samples were considered to differ in terms of that bin. The formula for calculating genomic identity was as follows: genomic identity = (total number of bins − number of divergent bins)/total number of bins × 100%.

### 2.3. Functional Gene Analysis

The SLYm1R high-density rice whole-genome SNP chip was used to rapidly detect important functional genes in ten *japonica* soft rice varieties from the Yangtze River Delta region. This chip enabled the detection of approximately 140 genes controlling key rice traits, including grain quality, yield, biotic and abiotic stress resistance, growth period, fertility, and plant architecture, and all the ten varieties were included in every functional gene analysis. Functional gene analysis included the following: SNP/INDEL analysis, haplotype analysis, and *Waxy* gene analysis. SNP/INDEL analysis revealed the existence or absence of functional genes by detecting genetic variations at their specific loci [10]. Haplotype analysis determined whether a sample contains a specific resistance gene according to a group of polymorphic markers within the target gene and its flanking 100 kb regions. *Waxy* gene analysis was performed to accurately analyze *Waxy* genotypes according to established methods [11].

## 3. Results

### 3.1. Genomic Homozygosity and Indica–Japonica Fragments in Major Japonica Soft Rice Varieties from the Yangtze River Delta Region

**Figure 1:**
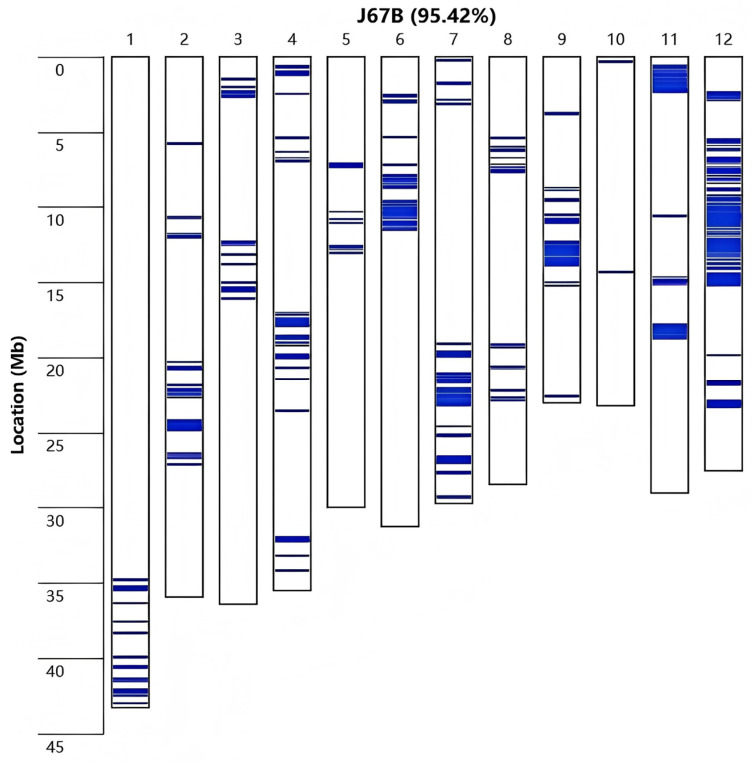
Genomic homozygosity of Jia 67. The *y*-axis represents chromosome position, and the *x*-axis shows chromosome names. Heterozygous loci are marked in blue on the plot.

**Figure 2:**
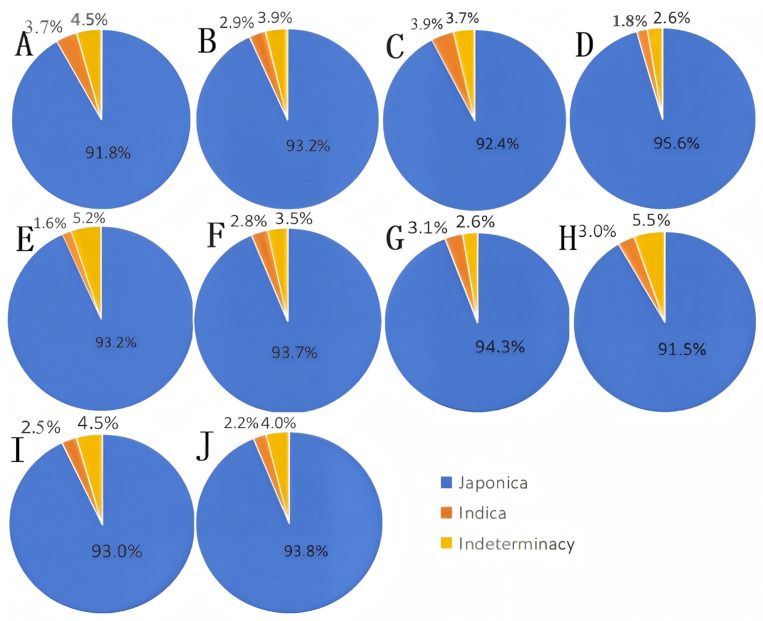
Analysis of *indica–japonica* genomic fragments in ten *japonica* varieties. Composition of *indica–japonica* genomic fragments in (**A**) Huruan 1212, (**B**) Meigu 2, (**C**) Songxiangjing 1018, (**D**) Tai’an 1, (**E**) Nanjing 46, (**F**) Nanjing 9108, (**G**) Nanjing 5055, (**H**) Zhehexiang 2, (**I**) Jia 58, and (**J**) Jia 67.

Quality control analysis of the SLYm1R SNP chip across the ten *japonica* soft rice varieties revealed a low average missing_ratio of 0.7% and a high call rate exceeding 90%, demonstrating robust reliability and accuracy of the chip-based genotyping (Table 2). To genetically confirm whether the sample population has achieved a high degree of genotypic uniformity, sample homozygosity was determined using high-quality loci obtained by screening whole-genome SNP chip data. The genomic homozygosity_ratio of Jia 67 was 95.4% (Figure 1), whereas nine *japonica* soft rice varieties, including Songxiangjing 1018, Meigu 2, Huruan 1212, Tai’an 1, Nanjing 46, Nanjing 9108, Nanjing 5055, Zhehexing 2, and Jia 58, all exhibited genomic homozygosity_ratio exceeding 99.9% (Supplementary Figure S1, Table 2). Gene chip fragment typing technology was used to analyze genomic segment origins, which revealed that 91.8%, 93.2%, 92.4%, 95.6%, 93.2%, 93.7%, 94.3%, 91.5%, 93.0%, and 93.8% of the genomic segments in Huruan 1212, Meigu 2, Songxiangjing 1018, Tai’an 1, Nanjing 46, Nanjing 9108, Nanjing 5055, Zhehexiang 2, Jia 58 and Jia 67, respectively, were from *japonica* rice (Figure 2A–I). This indicates that the soft rice varieties from the Yangtze River Delta region in China have a high proportion of *japonica* genomic components, all of which belong to typical *japonica* rice varieties. Among them, Tai’an 1 has the highest proportion of *japonica* components, while Zhehexiang 2 has the lowest.

Missing refers to the number of markers that failed genotyping. Missing_ratio denotes the percentage of markers that failed genotyping relative to the total number of markers. Marker_valid represents the number of valid markers. Heterozygosity indicates the number of heterozygous genotype markers. MG2: Meigu 2; SXJ1018: Songxiangjing 1018; TAIAN1: Tai’an 1; NG46: Nanjing 46; HR1212: Huruan 1212; NG5055: Nanjing 5055; NG9108: Nanjing 9108; D1A: Jia 58; ZHX2: Zhehexiang 2; J67B: Jia 67.

### 3.2. Clustering and Genomic Identity of Major Japonica Soft Rice Varieties in the Yangtze River Delta Region

**Figure 3:**
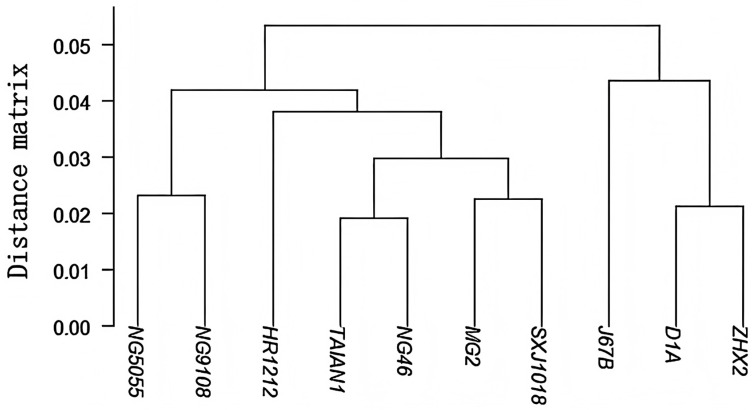
Cluster analysis of major *japonica* soft rice varieties in the Yangtze River Delta region.

**Figure 4:**
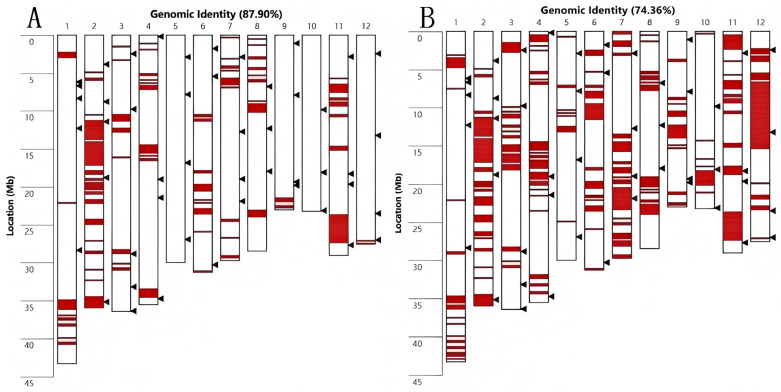
Genomic identity analysis of *japonica* soft rice varieties in the Yangtze River Delta region. (**A**) Genomic identity between Tai’an 1 and Nanjing 46. (**B**) Genomic identity between Tai’an 1 and Jia 67. The *y*-axis represents chromosome position, and the *x*-axis shows chromosome names. Red regions in the figure represent differential loci, and triangles indicate the positions of the 48 SSR markers specified in the Chinese National Standard (NY/T 1433-2014 technical specification for rice variety identification—SSR marker method).

A cluster analysis was conducted on the ten *japonica* soft rice varieties from the Yangtze River Delta region. As shown in Figure 3, when the aggregation level was set to 0.045, the ten varieties could be divided into two major groups. Seven rice varieties sourced from Jiangsu Province and Shanghai were classified into Group I, which covered Nanjing 5055, Nanjing 9108, Nanjing 46, Huruan 1212, Tai’an 1, Meigu 2 and Songxiangjing 1018. Group II consisted of three varieties from Zhejiang Province, including Jia 67, Jia 58 and Zhehexiang 2. Analysis of genomic identity indicated that Tai’an 1 and Nanjing 46 had the highest genomic identity (87.9% similar; Figure 4A), while Tai’an 1 and Jia 67 had the lowest genomic identity (74.4% similar; Figure 4B). Combined results of cluster analysis and genomic identity analysis revealed that *japonica* soft rice varieties from Shanghai shared a closer genetic relationship with Jiangsu varieties than with those from Zhejiang Province.

### 3.3. Functional Genes Associated with Important Agronomic Traits, Such as Grain Yield and Quality, in Japonica Soft Rice Varieties from the Yangtze River Delta Region

Based on the results of the chip assay, thirteen functional genes controlling key traits, such as yield, quality, and growth period, were identified in the ten analyzed varieties (Table 3). Among the identified yield-related genes, two were associated with increased grain width. More specifically, *GS5* [12] was detected in Songxiangjing 1018, Tai’an 1, Zhehexiang 2, Huruan 1212, Nanjing 46, Meigu 2, and Jia 67, whereas *GW5* [13] was detected in Nanjing 5055, Nanjing 9108, and Jia 58. All ten varieties carried *HESO1*, which is associated with late maturation [14]. The other identified late maturation-related genes were *Hd3b* [15], which was detected in all varieties except for Zhehexiang 2 and Jia 67, and *OsGATA28* [14], which was detected in Tai’an 1, Zhehexiang 2, and Jia 58. Interestingly, *Hd3a*, which is related to early maturation, was detected in Nanjing 5055, Nanjing 9108, and Jia 58. The diversity in heading dates among the examined varieties may be partly explained by differences in heading date-related gene combinations among genomes. In terms of quality-related genes, except for the three varieties, Huruan 1212, Meigu 2, and Jia 67, which showed no signal, *Wx^mp^* (associated with low amylose contents) [16] was detected in all other varieties, which is in accordance with their classification as soft rice varieties. With the exception of Huruan 1212 and Jia 67, the analyzed varieties carried the fragrance-related gene *Badh2* [17], implying that they are fragrant rice varieties.

### 3.4. Functional Genes Associated with the Biotic and Abiotic Stress Resistance of Japonica Soft Rice Varieties from the Yangtze River Delta Region

The SLYm1R high-density rice whole-genome SNP chip was also used to rapidly screen for genes conferring resistance to biotic and abiotic stresses in ten *japonica* soft rice varieties. thirteen functional genes associated with biotic and abiotic stress resistance were detected in the examined varieties (Table 4). In terms of biotic stress resistance genes, the broad-spectrum blast resistance gene *Pi2* [18] was detected in Zhehexiang 2 and Jia 58. The major broad-spectrum blast resistance gene *Pita* [19] was detected in Songxiangjing 1018, Zhehexiang 2, and Meigu 2. Another major blast resistance gene, *Pid3* [20], was detected in Huruan 1212. The bacterial blight resistance gene *Xa26/Xa3* was detected in Zhehexiang 2, Jia 58, and Huruan 1212. *STV11*, which confers durable resistance to the rice stripe virus, was detected in Nanjing 5055 and Nanjing 9108 [21]. Overall, there is an urgent need to improve the disease resistance of *japonica* soft rice in the Yangtze River Delta, especially regarding rice blast, as few current varieties carry multiple major resistance genes simultaneously. Of the identified abiotic stress resistance genes, *BET1*, *OsPP15*, *qUVR-10* [22], and *DRO1* were widely distributed among the analyzed varieties. Intriguingly, several abiotic stress resistance genes were detected in Nanjing 46 and Meigu 2, including *qLTG3-1* (cold tolerance) [23], *BET1* (boron tolerance), *OsPP15* (drought tolerance), *bZIP73* (cold tolerance) [24], and *NRAT1* (aluminum tolerance) [25].

### 3.5. Comparison of Main Agronomic Traits of Japonica Soft Rice Varieties in the Yangtze River Delta Region

The *Wx* gene exhibits pleiotropic effects, influencing not only amylose content but also significantly affecting gel consistency [26]. Rice varieties carrying the *Wx^mp^* allele typically exhibit an amylose content of approximately 10% [16] and relatively high gel consistency. The *japonica* soft rice varieties grown in the Yangtze River Delta region predominantly carry the *Wx^mp^* gene (Table 3), characterized by relatively low amylose content ranging from 8% to 14.5% and high gel consistency ranging from 69 mm to 90 mm (Table 5). Thus, most *japonica* soft rice varieties in this region produce grains ideal for eating (i.e., soft and elastic cooked rice with non-retrograded starch). Aroma, as one of the important sensory characteristics of rice, has a significant impact on the eating and cooking quality. The *BADH2* gene, located on chromosome eight, is the primary genetic regulator of rice aroma [27]. *Badh2* was detected in almost all of these varieties; the expression of this gene results in a pleasant aroma. Among the analyzed varieties, Songxiangjing 1018, Huruan 1212, Nanjing 46, Nanjing 9108, and Zhehexiang 2 were awarded gold prizes in the National High-Quality Rice Taste Quality Evaluation (*Japonica* Rice), making them excellent germplasm resources for improving the taste quality of premium rice varieties. Specifically, Tai’an 1 stands out for its early maturity, short stature, low chalkiness, and high gel consistency, demonstrating exceptional performance across growth duration, appearance, and taste. Nanjing 5055 exhibited the best appearance quality among the tested varieties, with a chalkiness degree of only 0.8. In contrast, Jia 67 presented the poorest appearance quality, recording the highest chalkiness degree. Zhehexiang 2 and Jia 58 were revealed to carry the broad-spectrum blast resistance gene *Pi2*. Notably, Zhehexiang 2 is a blast-resistant variety that produces grains with excellent eating quality, suggesting that it is a valuable germplasm resource for enhancing the blast resistance of high-quality rice varieties.

## 4. Discussion

Similarity assessments were categorized into genetic similarity and genomic identity. Genetic similarity only considered the ratio of differential markers to total detected markers. In contrast, the genomic identity analysis adopted in this study divided the rice genome into 378 bins of 1 Mb. If a SNP difference existed within a bin, the two samples were considered different in that bin. This avoided the error where discrete differences across the genome led to a misleadingly high genetic similarity, making genomic identity a better indicator of actual sample resemblance. In this study, *japonica* soft rice varieties grown in the Yangtze River Delta region of China were characterized by a high proportion of *japonica* genomic segments. Moreover, genomic identity analysis reveals that the genomes of the analyzed varieties were highly similar, especially the genomes of the *japonica* soft rice varieties from Shanghai and Jiangsu Province. The limited availability of genetic donors for soft rice may be the primary reason for this similarity. The soft rice gene *Wx^mp^*, which was detected in Nanjing 46, Nanjing 9108, and Nanjing 5055, was derived from the Japanese germplasm resource Kanto 194. Furthermore, high-quality *japonica* soft rice varieties from Shanghai, including Tai’an 1, were developed using Nanjing 46 as one of the parental lines [28]. *Japonica* soft rice varieties from Zhejiang exhibited a relatively more distant genetic relationship with those from Jiangsu and Shanghai. This is attributed to the fact that, although varieties from Zhejiang Province, such as Zhehexiang 2 and Jia 58, also carry the *Wx^mp^* allele, they trace their origin to Jia 06-64, which is an improved progeny of the American variety Rico No. 1 (low amylose content), resulting in a relatively diverse genetic background [29]. The relatively narrow genetic diversity of soft rice varieties has, to some extent, increased the vulnerability of rice to disease resistance and stress tolerance [30]. Under climate anomalies and emerging pests and diseases, this poses a threat to yield stability and food security. Therefore, it is imperative to explore novel soft rice genes and develop genetically diverse soft rice germplasms to broaden the genetic foundation of varieties. Genome-wide association studies (GWAS) could strengthen the identification and screening of diverse disease-resistant germplasm resources. Meanwhile, the integration of molecular marker-assisted selection (MAS) and genomic selection (GS) technologies will accelerate the breeding process of diverse *japonica* soft rice varieties.

Although *japonica* soft rice varieties produce high-quality grains suitable for eating, those that are widely promoted for large-scale cultivation are often highly susceptible to diseases, particularly rice blast, which has prevented the widespread cultivation of high-quality soft rice varieties [31]. Rice blast, which is one of the most devastating rice diseases, is caused by the ascomycetous fungus Magnaporthe grisea (Hebert) Barr. It is a common disease in all major rice-growing regions worldwide, leading to annual yield losses of 10–20%, with the associated economic losses amounting to billions of dollars [32]. In China, rice blast is prevalent in rice-growing regions in South China, Southwest China, Northeast China, and the middle and lower reaches of the Yangtze River, with an annual affected area ranging from 3.3 to 5.7 million hectares [33]. Among rice blast control measures, breeding and promoting blast-resistant varieties is considered to be the most economical and effective approach. The Jiangsu Lixiahe Institute of Agricultural Sciences used molecular breeding techniques to introduce the broad-spectrum blast resistance gene Pigm into the elite soft rice variety Nanjing 9108, thereby developing the blast-resistant *japonica* soft rice variety Jinxiangyu 1. Additionally, Nanjing Agricultural University combined multiple blast resistance genes, including Pita and Pi54, to breed the moderately blast-resistant *japonica* soft rice variety Ningjing 8. However, there are relatively few commercially cultivated blast-resistant *japonica* soft rice varieties. Aggregating major resistance genes to develop new blast-resistant *japonica* soft rice varieties remains a critical challenge for researchers. Pi2 on rice chromosome 6 belongs to the NBS-LRR gene class and encodes a protein that confers broad-spectrum resistance to most of the 792 rice blast pathogen races collected in China. Interestingly, only 7.55% of these races can infect the parental line C101A51 carrying Pi2 [34]. In addition, Pita, located near the centromeric region of rice chromosome 12, consists of two exons and one intron (1463 bp). It encodes a plasma membrane receptor protein comprising 928 amino acids. To exert its protective effects, it interacts with the effector protein encoded by the avirulence gene AVR-Pita in the rice blast fungus, thereby triggering a defense response [19]. Combining Pi2 and Pita reportedly confers effective resistance to rice blast [35]. In the current study, genetic analyses of major *japonica* soft rice varieties grown in the Yangtze River Delta region indicated that Zhehexiang 2 carries the blast resistance genes Pi2 and Pita, the low-amylose content gene Wx^mp^, and multiple biotic and abiotic stress resistance genes, including Xa26/Xa3, BET1, and OsPP15. Furthermore, the grains of this variety are considered excellent for eating. The findings of this study provide valuable insights into genetic resources and carrier materials potentially useful for precisely and efficiently improving and innovating *japonica* soft rice germplasm resources via whole-genome selection.

Rice bacterial blight, caused by the Gram-negative bacterium *Xanthomonas oryzae* pv. *oryzae*, is another critical disease affecting rice yield. This pathogen can infect rice plants at all growth stages and attack various organs. Typically, bacterial blight leads to yield losses of 10–20%, with severe cases decreasing yield by 70–80% or even 100%, posing a serious threat to agricultural and food security [36]. In China, bacterial blight primarily affects major rice-producing regions in East and Central China. Although *japonica* rice is typically more resistant to bacterial blight than *indica* rice, the prevalence of this disease has recently increased in *japonica*-growing areas of the lower Yangtze River region largely because of frequent typhoons and heavy rainfall, pathogen buildup, widespread cultivation of susceptible varieties, and considerable pathogen diversity. Breeding resistant varieties using major bacterial blight resistance genes remains the most economical and effective approach to control this disease. To date, approximately 49 bacterial blight resistance genes have been identified in rice, of which the following 18 have been cloned: *Xa1*, *Xa2/Xa31*, *Xa4*, *xa5*, *Xa7*, *Xa10*, *xa13*, *Xa14*, *Xa21*, *Xa23*, *Xa3/Xa26*, *Xa27*, *xa25*, *Xa27*, *xa41*, *xa44*, *xa45*, and *Xa48* [37]. These genes may be exploited by breeding programs to develop bacterial blight-resistant rice varieties. *Xa3/Xa26*, derived from the *indica* restorer line Minghui 63, encodes an LRR-RLK-type receptor kinase. It provides resistance to the Chinese bacterial blight pathotype JL691 and is widely used in Chinese rice breeding materials [38]. The resistance conferred by *Xa3/Xa26* exhibits a significant dose effect and is influenced by the rice genetic background, with stronger resistance in plants with a *japonica* genetic background than in plants with an *indica* genetic background [39]. This study revealed that the *japonica* soft rice varieties from the Yangtze River Delta region carried relatively few resistance genes to bacterial leaf blight, and only *Xa3/Xa26* was detected in Zhehexiang 2, Jia 58, and Huruan 1212. Moreover, due to factors such as the degradation of resistance conferred by the *Xa3* gene or the influence of the genetic background of the varieties, the bacterial blight resistance of varieties carrying this gene, such as Jia 58, has not been significantly enhanced. There is an urgent need to strengthen the molecular improvement of resistance to bacterial leaf blight in *japonica* soft rice varieties in the Yangtze River Delta region, especially by enhancing the molecular aggregation of *Xa3/Xa26* and other major-effect bacterial blight resistance genes.

Although the high-density SLYm1R SNP array provides a high-throughput, efficient, and cost-effective platform for genome-wide genotyping, its reliance on predefined probe sets imposes inherent limitations in capturing novel alleles, structural variations in unprobed regions, and rare mutations [40]. This constraint restricts the comprehensive identification and fine-mapping of functional loci in rice. Given this limitation, with the continuous decrease in whole-genome sequencing costs, future studies should integrate whole-genome resequencing (WGS) and multi-omics analyses to refine genotyping resolution for *japonica* soft rice.

## 5. Conclusions

Soft rice varieties cultivated in the Yangtze River Delta region had a high proportion of *japonica* genomic components and were all classified as typical *japonica* rice varieties. The Tai’an 1 genome was most similar and dissimilar to the genomes of Nanjing 46 (87.9% genomic identity) and Jia 67 (74.4% genomic identity), respectively. *Japonica* soft rice varieties in the Yangtze River Delta region were characterized by amylose contents ranging from 8% to 14.5%, primarily governed by the low-amylose gene *Wx^mp^*, coupled with high gel consistency. Most varieties carried the fragrance gene *Badh2* and exhibited superior eating quality. However, varieties pyramiding multiple major resistance genes for rice blast and bacterial blight were scarce. In the future breeding of *japonica* soft rice, it is necessary to strategically introduce genes related to disease resistance so as to achieve coordinated integration of quality and resistance.

## Figures and Tables

**Table 1:** Classification standards for distinguishing *indica* and *japonica* rice types according to the frequency of *indica* and *japonica* type genes [9].

Gene Frequency		The Identified Type of Cultivated Rice
*indica* Type	*japonica* Type	
>0.90	<0.10	Typical *indica*
0.75–0.89	0.11–0.25	*Indica*
0.61–0.74	0.26–0.39	Partial *indica*
0.40–0.60	0.40–0.60	Intermediate type
0.26–0.39	0.61–0.74	Partial *japonica*
0.11–0.25	0.75–0.89	*Japonica*
<0.10	>0.90	Typical *japonica*

**Table 2:** Genotyping quality control data for the SLYm1R SNP chip.

Varieties	Missing	Missing_Ratio/%	Call Rate /%	Marker_Valid	Heterzygosity	Homozygosity	Homozygosity_Ratio/%
SXG1018	152.0	0.5	99.5	31,551.0	0.0	31,551.0	100.0
MG2	361.0	1.2	98.8	29,153.0	0.0	29,153.0	100.0
HR1212	145.0	0.5	99.5	31,558.0	0.0	31,558.0	100.0
TAIAN1	114.0	0.4	99.6	31,589.0	0.0	31,589.0	100.0
NG46	219.0	0.7	99.3	29,295.0	0.0	29,295.0	100.0
NG9108	288.0	0.9	99.1	31,415.0	0.0	31,415.0	100.0
NG5055	293.0	0.9	99.1	31,410.0	0.0	31,410.0	100.0
ZHX2	161.0	0.5	99.5	31,542.0	2.0	31,540.0	100.0
D1A	295.0	0.9	99.1	31,408.0	18.0	31,390.0	99.9
J67B	268.0	0.9	99.1	29,246.0	1340.0	27,906.0	95.4
Average	229.6	0.7	99.3	30,816.7	136.0	30,680.7	99.5

**Table 3:** Functional genes related to important agronomic traits of ten *japonica* soft rice varieties.

Gene	MSU ID	Chr	Alt_ Allele Function	SXJ1018	TAIAN1	ZHX2	HR1212	NG46	MG2	NG5055	NG9108	D1A	J67B
*GW5*	LOC_Os05g09520	5	Enhancing grain width							√	√	√	
*GS5*	*LOC_Os05g06660*	5	Enhancing grain width	√	√	√	√	√	√				√
*Wx^mp^*	LOC_Os06g04200	6	Low amylose content	√	√	√	N	√	N	√	√	√	N
*Badh2*	LOC_Os08g32870	8	Aroma	√	√	√		√	√	√	√	√	
*OsAAP6*	LOC_Os01g65670	1	Increasing seed protein content	√	√	√	√	√	√				
*Hd3b*	LOC_Os06g05060	6	Later days to headings	√	√	√		√	√	√	√	√	
*HESO1*	LOC_Os01g62780	1	Later days to headings	√	√	√	√	√	√	√	√	√	√
*OsGATA28*	LOC_Os11g08410	11	Later days to headings		√	√						√	
*Hd3a*	*LOC_Os06g06320*	6	Promoting heading							√	√	√	
*Rc*	*LOC_Os07g11020*	7	Red seed coat	√	√	√	√						
*qSH1*	LOC_Os01g62920	1	Seed shattering	√	√		√	√	√				√
*BOC1*	*LOC_Os03g12820*	3	Reducing callus browning					√	√				√
*TAC1*	*LOC_Os09g35980*	9	Reducing the tiller angle					√	√				√

“√” indicates that the actual sample phenotype was consistent with the phenotype described in the “Alt_Allele Function” column. N: no signal detected.

**Table 4:** Functional genes related to the biotic and abiotic stress resistance of ten *japonica* soft rice varieties.

Gene	MSU ID	Chr	Alt Allele Function	SXG1018	TAIAN1	ZHX2	HR1212	NG46	MG2	NG5055	NG9108	D1A	J67B
*Xa26/Xa3*	LOC_Os11g47210	11	Enhancing resistance to bacterial blight			√	√			N	N	√	
*Pita*	LOC_Os12g18360	12	Blast resistance	√		√			√				
*Pi2*	LOC_Os06g17900	6	Blast resistance			√						√	
*Pid3*	LOC_Os06g22460	6	Blast resistance				√						
*STV11*	LOC_Os11g30910	11	Durable resistance to rice stripe virus							√	√		
*qLTG3-1*	LOC_Os03g01320	3	Cold resistance					√	√	√	√	√	√
*BET1*	LOC_Os04g40140	4	Increasing boron-toxicity tolerance	√	√	√	√	√	√				√
*OsPP15*	LOC_Os01g62760	1	Increasing drought tolerance	√	√	√	√	√	√				√
*qUVR-10*	LOC_Os10g08580	10	High CPD photolyase activity	√	√	√	√			√	√	√	
*bZIP73*	LOC_Os09g29820	9	Enhancing cold tolerance					√	√				√
*NRAT1*	LOC_Os02g03900	2	Enhancing aluminum toxicity tolerance					√	√				√
*OsSAP16*	LOC_Os07g38240	7	Improving seed germination rate under low-temperature conditions	√			√			N	N	N	
*DRO1*	LOC_Os09g26840	9	Increasing root depth and drought tolerance	√	√	√	√	√	√				√

“√” indicates that the actual sample phenotype was consistent with the phenotype described in the “Alt_Allele Function” column. N: no signal detected.

**Table 5:** Performance of main agronomic traits of ten major *japonica* soft rice varieties in the Yangtze River Delta region.

Varieties	Whole Growth Period/D	Plant Height/cm	Blast Resistance	Bacterial Blight Resistance	Whole-Head Rice Rate/%	Chalk-Iness/%	Gel Consistency/mm	Amylose Content/%
SXG1018	161.2	99.8	MR	MS	70	3.1	76	9.1
MG2	159.2	99.1	MS	-	72.8	1.1	83	11.5
HR1212	156	107.4	S	-	70.3	2.1	86	8.3
TAIAN1	149.1	98.4	MS	-	72.5	2.3	80	8.4
NG46	164.5	110.1	MS	MS	66.8	2.4	83	10.6
NG9108	153	96.4	S	MS	71.4	3.1	90	14.5
NG5055	160	96.5	S	MS	71.4	0.8	87	10.1
ZHX2	167.6	102.1	R	-	65	1.5	81	11.2
D1A	156	100.3	R	MS	72	8.5	69	10.0
J67B	160	94.1	MS	MR	72.9	11.9	85	8.5

The presented information was sourced from the National Rice Data Center (https://www.ricedata.cn/variety/, accessed on 17 May 2026). R: resistant; MR: moderately resistant; MS: moderately susceptible; S: susceptible.

## Data Availability

The original contributions presented in this study are included in the article and Supplementary Materials. Further inquiries can be directed to the corresponding author.

## Supplementary Materials

The following supporting information can be downloaded at: https://www.mdpi.com/article/10.3390/cimb48070738/s1.
