# Supplementary material for: Genotypic Characterization and Evaluation of Japonica Soft Rice Varieties in the Yangtze River Delta Region of China
Source: Curr Issues Mol Biol. 2026 Jul 20;48(7):738. doi: 10.3390/cimb48070738 (PMC13409563; doi:10.3390/cimb48070738)
Supplement: Supplementary file 1 [file cimb-48-00738-s001.zip › cimb-4417035-supplementary.pdf]

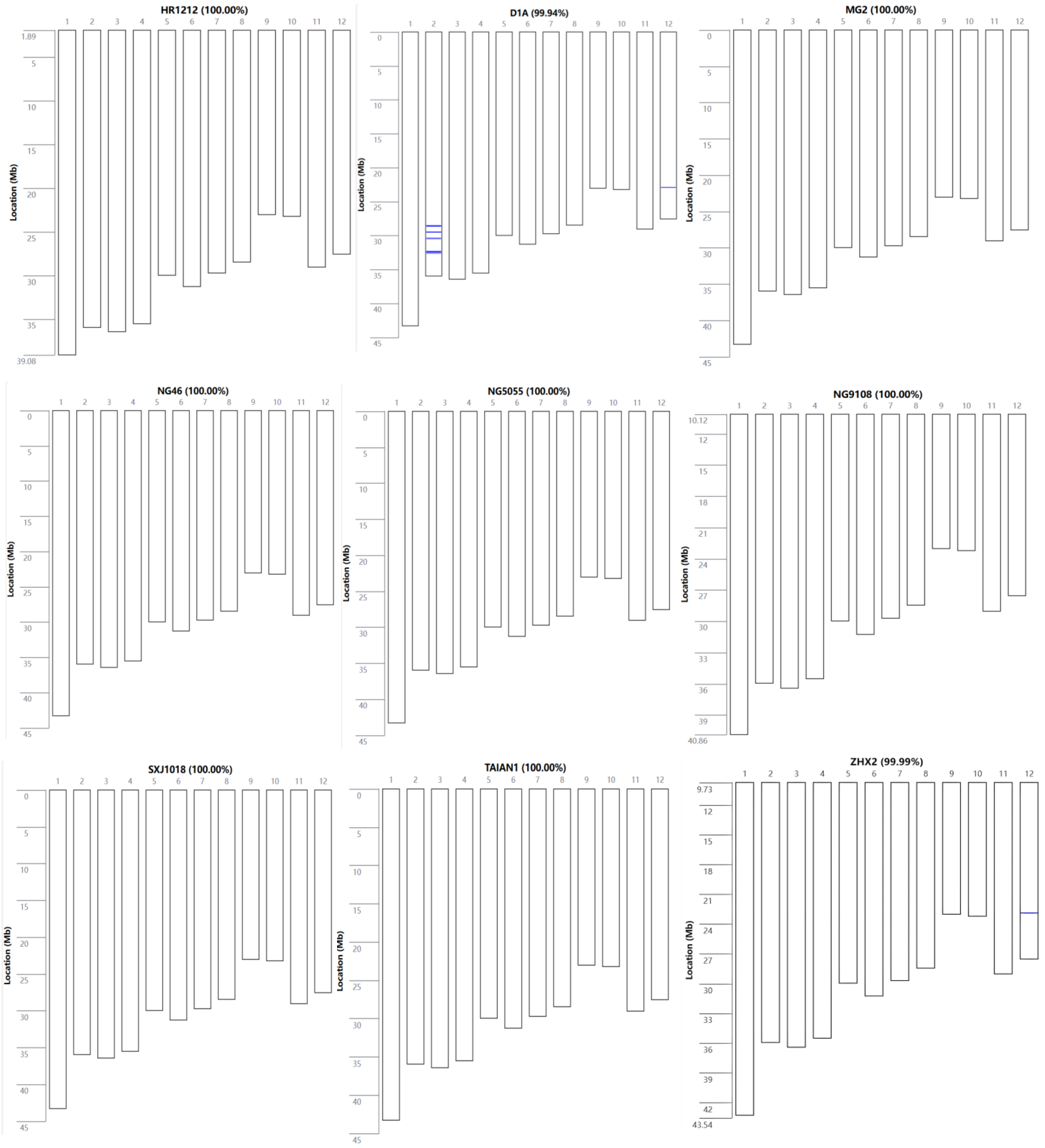

Supplementary Figure S1. Genomic homozygosity of nine major *japonica* soft rice varieties from the Yangtze River Delta region. The y-axis represents chromosome length (Mb), and the x-axis shows chromosome names. Heterozygous loci are marked in blue on the plot. MG2: Meigu 2; SXJ1018: Songxiangjing 1018; TAIAN1: Tai'an 1; NG46: Nanjing 46; HR1212: Huruan 1212; NG5055: Nanjing 5055; NG9108: Nanjing 9108; D1A: Jia 58; ZHX2: Zhehexiang 2.
